# Connecting Structural Characteristics and Material Properties in Phase-Separating Polymer Solutions: Phase-Field Modeling and Physics-Informed Neural Networks

**DOI:** 10.3390/polym15244711

**Published:** 2023-12-14

**Authors:** Le-Chi Lin, Sheng-Jer Chen, Hsiu-Yu Yu

**Affiliations:** Department of Chemical Engineering, National Taiwan University, No. 1, Sec. 4, Roosevelt Rd., Taipei 10617, Taiwan; d12524005@ntu.edu.tw (L.-C.L.); d12524004@ntu.edu.tw (S.-J.C.)

**Keywords:** Cahn–Hilliard equation, physics-informed neural network, Flory–Huggins parameter, membrane design, phase separation

## Abstract

The formed morphology during phase separation is crucial for determining the properties of the resulting product, e.g., a functional membrane. However, an accurate morphology prediction is challenging due to the inherent complexity of molecular interactions. In this study, the phase separation of a two-dimensional model polymer solution is investigated. The spinodal decomposition during the formation of polymer-rich domains is described by the Cahn–Hilliard equation incorporating the Flory–Huggins free energy description between the polymer and solvent. We circumvent the heavy burden of precise morphology prediction through two aspects. First, we systematically analyze the degree of impact of the parameters (initial polymer volume fraction, polymer mobility, degree of polymerization, surface tension parameter, and Flory–Huggins interaction parameter) in a phase-separating system on morphological evolution characterized by geometrical fingerprints to determine the most influential factor. The sensitivity analysis provides an estimate for the error tolerance of each parameter in determining the transition time, the spinodal decomposition length, and the domain growth rate. Secondly, we devise a set of physics-informed neural networks (PINN) comprising two coupled feedforward neural networks to represent the phase-field equations and inversely discover the value of the embedded parameter for a given morphological evolution. Among the five parameters considered, the polymer–solvent affinity is key in determining the phase transition time and the growth law of the polymer-rich domains. We demonstrate that the unknown parameter can be accurately determined by renormalizing the PINN-predicted parameter by the change of characteristic domain size in time. Our results suggest that certain degrees of error are tolerable and do not significantly affect the morphology properties during the domain growth. Moreover, reliable inverse prediction of the unknown parameter can be pursued by merely two separate snapshots during morphological evolution. The latter largely reduces the computational load in the standard data-driven predictive methods, and the approach may prove beneficial to the inverse design for specific needs.

## 1. Introduction

Phase separation is a spontaneous process triggered by the entry to an unstable region in the phase diagram. In the process, the initially homogeneous system demixes into two coexisting phases, corresponding to compositions that are either rich or poor in one of the components. The two phases are separated by distinct boundaries that change with time, thus resulting in morphology evolution during phase separation. The formed morphology impacts the material properties fundamentally, thus making the understanding of the phase separation process and the prediction of the resulting structures imperative [1,2,3,4,5,6]. For instance, polymer membranes are ubiquitously applied in the separation process in engineering and industries [7,8]. The preparation methods are commonly based on phase inversion and separation, including nonsolvent-induced phase separation (NIPS), vapor-induced phase separation (VIPS), or temperature-induced phase separation (TIPS) [9,10]. In a standard procedure, the polymer-rich phase grows with time and eventually forms the main structure of the membrane by solidification, while the polymer-poor phase becomes pores after post-treatment [11]. Therefore, it is essential to identify the key factors that impact phase separation dynamics of membrane-forming polymers and use the resultant to inversely design the membrane constituents to meet the required permeability, selectivity, and mechanical strength in different applications [12,13]. This study aims to synergistically connect the morphological characteristics in a phase-separating binary solution to the inherent physical properties. Two distinct approaches, continuum phase-field modeling, and physically driven neural network algorithm, are pursued.

The morphology that emerges during the phase separation process is inherently influenced by a combination of carefully selected materials and the specific physical conditions employed. Empirical experimentation involving testing a vast number of materials, various compositions within these materials, and diverse processing conditions is often deemed time-consuming and resource-intensive. Several theoretical approaches and simulation methods have been developed to predict phase evolution dynamics systematically and efficiently. Among them, the phase-field theory with the Cahn–Hilliard equation stands out as a popular tool for describing the evolution of morphology [14]. The model accounts for the interfacial energy between different phases through variational formulations, guiding the system toward equilibrium by minimizing the interfacial energy by aggregating the same phases. The versatility of the Cahn–Hilliard model has made it a valuable tool for studying various systems, including polymeric multicomponent systems under different temperatures and compositions [15], those involving dynamic flows [16], bubble motion [17], elastic materials [18], and more [19,20,21]. However, it is worth noting that investigations into the impact of system parameters on the resulting morphology have often been conducted separately, with only a few comprehensive studies comparing the relative influence of the parameters [22,23,24]. Since morphology is directly affected by system parameters encompassing material properties and applied conditions, effective morphology design should be grounded in a thorough understanding of the weight or significance of each parameter. Identifying the most influential system parameter, which we term the “morphology-determining parameter”, can provide valuable insights and guide a more directed approach to the overall membrane design.

Research efforts have substantially relied on extensive forward simulations to predict the phase separation patterns by inputting various sets of system parameters. However, the inherent complexity of morphological evolution has left us lacking a unified explanation for the phase separation phenomenon [25,26,27]. Some studies have even highlighted the challenges of simulating the Cahn–Hilliard equation, attributed to its high-order derivatives, which place significant demands on computational resources and pose challenges in maintaining numerical stability [28,29]. In light of these challenges and the rapid advancements in neural network algorithms, there has been a remarkable surge in studies leveraging the capability of neural networks for conducting inverse simulations [30,31,32,33,34,35,36,37]. These inverse simulations aim to uncover the unknown properties associated with desired morphologies, effectively reversing the traditional approach.

Notably, the physics-informed neural network (PINN) has gained prominence in recent years for this purpose. The PINN, characterized by its numerous network parameters, is a robust and versatile universal function approximator capable of representing a wide range of theory-guided equations [38,39,40]. In traditional forward simulations, the PINN determines the network parameters that best approximate the target equation by minimizing both the residual loss and constraints that adhere to the governing physical laws without relying on experimental or simulation data [41,42,43]. Conversely, in inverse simulation, the PINN is capable of finding concealed knowledge from a small amount of experimental or simulation data by minimizing the residual loss and data loss [44,45]. In regard to applying the inverse discovery to the phase-field problem, ref. [37] has developed an advanced PINN approach that utilizes the pseudo-spectral discretization in space to handle the morphology changes from image snapshots with a corresponding loss function constructed through a stabilized time-stepping scheme. The free energy function of a simple bistable form has been inversely predicted. Aside from this study, relatively few efforts have been devoted specifically to the inverse simulation of the Cahn–Hilliard phase separation, particularly in employing the PINN framework to discover the unknown parameters underlying desired morphologies.

In this study, we focus on the morphology design problem, specifically delving into the membrane microstructure within a 2D polymer solution system. The Cahn–Hilliard equation incorporated with the Flory–Huggins free energy model [46] serves as the system descriptor to characterize the system. Our approach begins with an in-depth analysis of the morphology properties, employing methods such as the self-correlation function and the Minkowski functionals [47,48,49]. Through this analysis, we elucidate the impact of each system parameter on the resulting morphology. Subsequently, a comprehensive sensitivity analysis is achieved to discern the relative importance or weight of each system parameter within our specific context. This analysis leads us to identify the morphology-determining parameter, a critical insight for effective morphology design. After that, we take an exemplary attempt at inverse simulations to uncover the morphology-determining parameter from a set of given morphology snapshots. The connection between the morphology characteristics and the embedded parameter is accomplished through a renormalization approach.

## 2. Methods and Analysis

### 2.1. Numerical Pseudo-Spectral Method

In this work, the evolving morphology in 2D is described by the Cahn–Hilliard model [50] for the spatial and temporal variations of the substance,

(1) $$\frac{\partial \phi_{i}}{\partial t}=\nabla \cdot M\nabla \mu_{i},$$

(2) $$\mu_{i}=\frac{\delta F}{\delta \phi_{i}},$$

(3) $$F=\int (f\left(\phi_{i}\right)+\kappa {\left(\nabla \phi_{i}\right)}^{2})\;dA,$$

where $\phi_{i}$ represents the volume fraction of the substance *i*, $\mu_{i}$ is its chemical potential, M is the mobility, κ is the surface tension parameter, A is the total surface area, F is the total free energy, and f  is the free energy density. The mobility may be concentration-dependent due to inter-species diffusion but is generally assumed as a constant in literature [15,16,49,51]. Moreover, although different formulations of mobility change the scaling of the domain growth law, the morphological features are not significantly impacted [52]. We assume a constant mobility to more transparently elucidate the effect of each parameter in the system. The surface tension parameter characterizes the contribution to the system free energy from the concentration gradient at the interface. It can be viewed as a penalty term in addition to the free energy of a homogeneous system. Similarly, we neglect its concentration dependence and assume a constant value of κ consistent with phase-separating polymer systems [51,53]. In a binary system containing polymer and solvent, the Flory–Huggins theory [46] defines the free energy density as

(4) $$f=\frac{RT}{v_{0}}\left[\frac{\phi }{m_{p}}ln\phi +\frac{\left(1-\phi \right)}{m_{s}}\ln\left(1-\phi \right)+\chi_{FH}\phi \left(1-\phi \right)\right],$$

where $\phi_{i}=\phi$ is the polymer volume fraction, 1−ϕ is that for solvent, $\chi_{FH}$ represents the interaction parameter between the two substances, R is the gas constant, T is the temperature, $v_{0}$ is the volume of one segment unit, $m_{p}$ denotes the degree of polymerization of the polymer, and $m_{s}$ is the number of segments per solvent. The solvent is fixed as $m_{s}=1$, which represents the occupation of one unit volume in the polymer solution system. 

We conduct a pseudo-spectral algorithm to solve the Cahn–Hilliard equation incorporated with the Flory–Huggins free energy model described above in a square periodic domain in x and y with A=L×L. As shown in Equation (5), the pseudo-spectral algorithm comprises a Fourier-transformed volume fraction in space and an implicit Euler stepping in time,

(5) $$\frac{{\hat{\phi }}^{n+1}-{\hat{\phi }}^{n}}{\Delta t}=-q^{2}M{\hat{\mu }}^{n+1},$$

where the forward and inverse transforms are defined as

(6) $$\hat{\phi }\left(q_{x},q_{y}\right)=\sum_{j_{x}=0}^{N_{x}-1}\sum_{j_{y}=0}^{N_{y}-1}\phi \left(x_{j_{x}},y_{j_{y}}\right)e^{-i\left(j_{x}\Delta xq_{x}+j_{y}\Delta yq_{y}\right)}$$

and

(7) $$\phi \left(x,y\right)=\frac{1}{N_{x}N_{y}}\sum_{l_{x}=0}^{N_{x}-1}\sum_{l_{y}=0}^{N_{y}-1}\hat{\phi }\left(q_{x},q_{y}\right)e^{i\left(j_{x}\Delta xq_{x}+j_{y}\Delta yq_{y}\right)},$$

respectively. Here, $i=\sqrt{-1}$, $q_{x}=\frac{2\pi l_{x}}{L}$ and $q_{y}=\frac{2\pi l_{y}}{L}$ are the two wave numbers in x and y with $q^{2}=q_{x}^{2}+q_{y}^{2}$. $\Delta x=L/N_{x}$ and $\Delta y=L/N_{y}$ are spatial resolutions, and $N_{x}$ and $N_{y}$ are the number of grids in the two dimensions. The spatial indices are defined by $0{\leq j}_{x}\leq N_{x}-1$ and $0{\leq j}_{y}\leq N_{y}-1$ in real space and $0\leq l_{x}\leq N_{x}-1$ and $0\leq l_{y}\leq N_{y}-1$ in the Fourier space. The determination of the Fourier-transformed chemical potential $\hat{\mu }$ relies on evaluating the local volume fraction in real space; therefore, frequent forward and inverse transformations are required in numerical iterations. Δt is the time step size, and n is the number of time steps. 

We choose the system parameters consistent with ref. [51] summarized in Table 1. The Asterisked variables are physical quantities in real units. By selecting the unit of length as L, the dimensionless mobility and surface tension parameter are defined as $M=\frac{M^{*}RT^{*}t^{*}}{{L^{*}}^{2}{\nu_{0}}^{*}}$=1 and $\kappa =\frac{{\nu_{0}}^{*}\kappa^{*}}{RT^{*}{L^{*}}^{2}}=7.63\times 10^{-5}$. The phase separation would occur spontaneously based on the decomposition curve in the phase diagram obtained from [51]

(8) $$\chi_{c}=\frac{1}{2}\left[\frac{1}{m_{p}\phi }+\frac{1}{m_{s}\left(1-\phi \right)}\right],$$

where $\chi_{c}$ represents the critical interaction parameter and equals 2.08 for the reference case of $\phi_{0}=0.4$. A system with a $\chi_{FH}$ lower than 2.08 remains stable and would not trigger the phase separation. The dimensionless spatial domain has a length of unity with a discretization of 256 × 256 grids, and the dimensionless time step size is $\Delta t=4\times 10^{-9}$. The calculation is initialized with volume fraction fluctuations of a magnitude of 0.005. The predicted morphology evolutions given different parameters allow for a comprehensive investigation of the importance of each factor. Moreover, the simulated snapshots will be used as inputs for the neural network training.

### 2.2. Physics-Informed Neural Networks

In order for the PINN to learn the temporal variation during phase separation, two snapshots of morphologies taken at two distinct time frames, $t_{0}$ and $t_{1}$, are utilized as the training data. A successful PINN design relies on carefully considering possible difficulties while optimizing network parameters. In this regard, we introduce simple variations to improve the overall performance of the neural network. First of all, a common practice in PINN training is to renormalize the range of the data points in a similar order, and it is known that the neural network fails to train with unbalanced scales of input data [54,55]. In the numerical simulations, the dimensionless spatial domain considered in both the x- and y-directions is 0 to 1. However, the chosen time frames for training are arbitrary in general, and the range of the time interval may be quite distinct from that of the spatial domain. As will be demonstrated in the examples in Section 3.3, the chosen temporal interval for training is of the order of $10^{-3}$. To make all the variables of a similar range, we first shift the spatial domain to [−0.5, 0.5] in each direction. We then enlarge the scale of the time interval by 1000 times such that the time interval is in a similar range to the spatial domain. To accommodate the variation in the time interval, the mobility constant is downscaled 1000 times to compensate for the resulting influence in the integration of the Cahn–Hilliard equation. Such a mathematical rescaling may be adjusted according to the particular range of data selected in the PINN training, making the selection of the temporal interval flexible.

In the early stage of spinodal decomposition, the polymer concentration fluctuates in small amplitude and length scale. As a result, the PINN training becomes highly demanding as a large number of domain sampling points are required for the network parameters to capture the subtle variations. Moreover, the noisy decomposition pattern often results in flat output solutions from the PINN training, where the network parameters fail to be updated due to vanishing gradients [56] and converge to a spurious local minimum [57]. However, in the later coarsening stage, the amplitude of the fluctuation maintains a consistent scale, and the polymer-rich domains grow gradually. We intentionally select morphology snapshots during the coarsening stage and perform a PINN inverse design to avoid trivial PINN solutions.

The complex morphology patterns during the coarsening stage and the high-order derivatives in the Cahn–Hilliard equation make it challenging to train the neural networks and minimize the corresponding prediction error. Inspired by refs. [58,59,60], we propose a coupled structure, where one neural network (Model I) represents the concentration profile of the polymer and the other (Model II) represents the chemical potential of the polymer. The coupled PINN eases the burden of each model by reducing the amount of fed information and reducing the order of derivatives into a second-order differential problem for each model. Furthermore, an integrated sampling method [59] is employed to provide minimal sampling points while simultaneously maintaining the amount of fed information to the models. Details of the PINN algorithm are discussed as follows.

**Figure 1:**
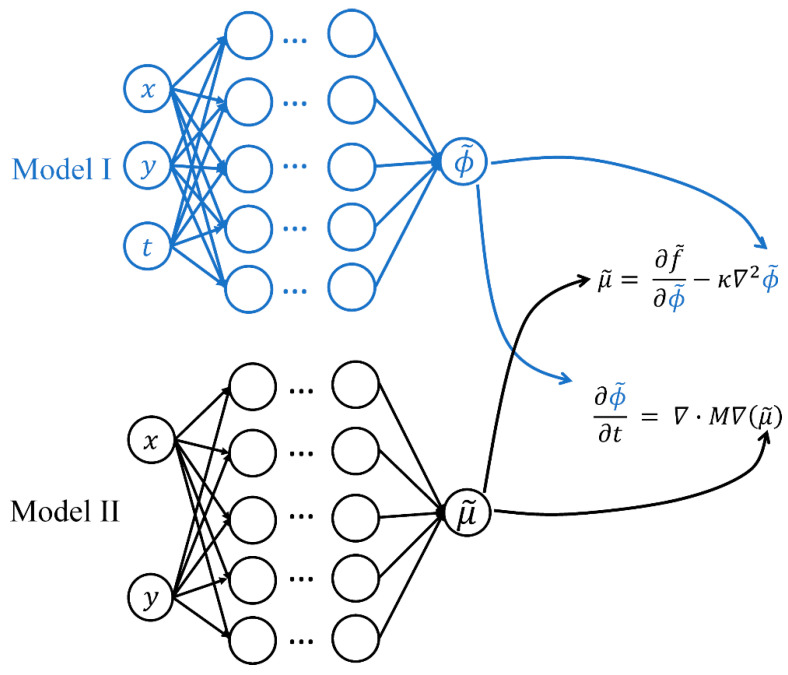
The coupled PINN has two models. Model I approximates the polymer volume fraction ($\tilde{\phi }$), and Model II approximates the chemical potential ($\tilde{\mu }$). Both models have six hidden layers and 128 nodes per layer. For each neuron, the sigmoid activation function takes the weights and biases from the previous layer, and an automatic differentiation (AD) algorithm is applied to deal with the derivatives in the PDE. We use Adam optimizer to update the network parameters to minimize the loss function.

As shown in Figure 1, the PINN structure consists of two fully connected feedforward neural networks, Model I and Model II, each with six hidden layers and 128 nodes in each layer. Model I takes three variables (x, y, and t) as input and outputs the polymer volume fraction ($\tilde{\phi }$). In contrast, Model II has two input variables (x and y), and the output variable is the chemical potential ($\tilde{\mu }$) evaluated for a given polymer distribution. The output variables with a tilde represent model predictions. The two models simultaneously minimize the loss function J defined as

(9) $$J=J_{PDE}+J_{BC}+100J_{t_{0}}+100J_{t_{1}}+10J_{\mu }.$$

Each loss term represents the mean square error during training, where the residual or PDE loss of the Cahn–Hilliard equation reads

(10) $$J_{PDE}:\frac{1}{N_{PDE}}\sum_{i=1}^{N_{PDE}}{\left|\frac{\partial \tilde{\phi }\left(x_{PDE}^{i},y_{PDE}^{i},t_{PDE}^{i}\right)}{\partial t}-\nabla \cdot M\nabla \tilde{\mu }\left(x_{PDE}^{i},y_{PDE}^{i}\right)\right|}^{2},$$

the periodic boundary loss,

(11) $$J_{BC}:\frac{1}{N_{BC}}\sum_{i=1}^{N_{BC}}{\left|\tilde{\phi }\left(-0.5,y_{BC}^{i},t_{BC}^{i}\right)-\tilde{\phi }\left(0.5,y_{BC}^{i},t_{BC}^{i}\right)\right|}^{2}\;\;\;\;\;\;\;\;\;\;\;\;+\frac{1}{N_{BC}}\sum_{i=1}^{N_{BC}}{\left|\tilde{\phi }\left(x_{BC}^{i},-0.5,t_{BC}^{i}\right)-\tilde{\phi }\left(x_{BC}^{i},0.5,t_{BC}^{i}\right)\right|}^{2},$$

the data loss at two time frames (snapshots),

(12) $$J_{t_{0}}:\frac{1}{N_{t}}\sum_{i=1}^{N_{t}}{\left|\tilde{\phi }\left(x_{t_{0}}^{i},y_{t_{0}}^{i},t_{0}\right)-\phi \left(x_{t_{0}}^{i},y_{t_{0}}^{i},t_{0}\right)\right|}^{2}$$

and

(13) $$J_{t_{1}}:\frac{1}{N_{t}}\sum_{i=1}^{N_{t}}{\left|\tilde{\phi }\left(x_{t_{1}}^{i},y_{t_{1}}^{i},t_{1}\right)-\phi \left(x_{t_{1}}^{i},y_{t_{1}}^{i},t_{1}\right)\right|}^{2},$$

and the mean square error between the chemical potential evaluated from the output of Model I and the predicted chemical potential of Model II,

(14) $$J_{\mu }:\frac{1}{N_{PDE}}\sum_{i=1}^{N_{PDE}}{\left|\tilde{\mu }\left(x_{PDE}^{i},y_{PDE}^{i}\right)-\frac{\partial \tilde{f}}{\partial \tilde{\phi }\left(x_{PDE}^{i},y_{PDE}^{i},t_{PDE}^{i}\right)}\;\;\;\;\;+\kappa \nabla^{2}\tilde{\phi }(x_{PDE}^{i},y_{PDE}^{i},t_{PDE}^{i})\right|}^{2}$$

with

(15) $$\tilde{f}=\frac{\tilde{\phi }}{m_{p}}ln\tilde{\phi }+\left(1-\tilde{\phi }\right)\ln\left(1-\tilde{\phi }\right)+\chi_{FH}\tilde{\phi }\left(1-\tilde{\phi }\right)$$

being the scaled Flory–Huggins equation.

In Equation (9), each loss term is weighted differently. We choose the weights for the PDE and the boundary losses as 1. Given this choice, the weight of the data loss in the loss function is the largest (set as 100) to ensure the model learns the two snapshots thoroughly and preliminarily. Since Model II is not subjected to additional constraints that govern its output, the weight of the chemical potential loss is enlarged modestly to make the two models evolve in a consistent manner. The results of a comparative analysis using different sets of weights are provided in Table S1 and Figure S1. In the analysis, the unknown parameter ($\chi_{FH}$) gradually converges to a constant value. Our current weight choice in Equation (9) yields the best performance.

Next, we explain our sampling method for each loss function. For the $J_{PDE}$ and $J_{\mu }$ sampling procedure, our approach involves two distinct sets of collocation points. Initially, we sample a total of 8192 collocation points uniformly, and these points remain constant throughout the entire training process. Additionally, for each epoch during training, we sample an additional 32 collocation points uniformly. To ensure comprehensive model robustness, we combine both the initially-sampled and newly-sampled collocation points and train them collectively during each epoch ($N_{PDE}=8224$). This strategy combines the stability of a large, fixed set of points with the adaptability gained by refreshing a smaller set of points in each epoch, ultimately enhancing the learnability of the model. For $J_{t_{0}}$, we adopt a similar approach. The initial data set at $t_{0}$ is selected using the Latin hypercube sampling method (LHS) [59], yielding 128 data points. Subsequently, for each epoch, we sample an additional 32 data points uniformly ($N_{t}=160$). The choice of the LHS method, known for its superior distribution uniformity [61] compared to a typical uniform sampling, is employed to enhance training robustness, especially when learning pure spatial distribution information. The procedure for $J_{t_{1}}$ mirrors that of $J_{t_{0}}$. In contrast, for $J_{BC}$, we initially sample 128 data points uniformly and maintain the same set of sampling points throughout the training process without renewal ($N_{BC}=128$). Finally, for each neuron, the activation function is the sigmoid function that takes the linear transformation of the output from the previous layer, which aligns with ref. [35]. The optimization strategy employs the Adam optimizer [62] with a learning rate set at $10^{-3}$. The PINN training process terminates after 700,000 epochs, with the loss function minimized to an order of $10^{-2}$. A quantitative comparative analysis of the training results for different learning rates and numbers of sample points are presented in Figures S2–S4. It can be seen from Figure S2 that choosing a learning rate that is too small ($10^{-4}$) or too large ($10^{-2}$) may lead to unsatisfactory predictions. In addition, Figures S3 and S4 suggest that the convergence of PINN is generally improved by increasing the numbers of the initial collocation points in the domain and the renewal sample points in each epoch. It should be noted that using more sample points generally results in a longer training time. Our selected numbers of sample points balance the satisfactory predictions and computational effort.

### 2.3. Morphology Analysis

We introduce geometrical and statistical descriptors to characterize the morphological evolution. The Minkowski functionals are a set of mathematical measures for analyzing the morphology and topology in a system [47,48,49,63]. Among the three geometrical characteristics, i.e., area fraction, boundary length, and domain connectivity, the connectivity correlates with pattern complexity, and we choose it as one of the crucial measures during phase separation. The connectivity is determined by the 2D Euler characteristic χ defined as

(16) $$\chi =\frac{1}{2\pi }\int_{\;}^{\;}\left[\frac{1}{r_{c}}\right]dc,$$

where c is the circumference and $r_{c}$ is the radius of the local curvature for the phase of interest. χ calculates the number of isolated polymer-rich areas in the system. In order to locate the phase boundary that separates the polymer-rich and polymer-poor phases, a threshold volume fraction, $\phi_{th}$, is specified. A compositional indicator function $I\left(x,y\right)$ is defined based on $\phi_{th}$. Through binarization, $I\left(x,y\right)=1$ if $\phi \left(x,y\right)\geq \phi_{th}$ whereas $I\left(x,y\right)=0$ otherwise. The choice of the threshold value impacts the determination of the phase domains [23]. Here, we choose $\phi_{th}=\phi_{0}+0.01$ to make the threshold value slightly larger than the initial condition. On this basis, we characterize how regions of the same phases are distributed in the system through the self-correlation function at a given time,

(17) $$S_{2}\left(r\right)=\langle I\left(x_{1},y_{1}\right)I(x_{2},y_{2})\rangle ,$$

where $r=\sqrt{{\left(x_{2}-x_{1}\right)}^{2}+{\left(y_{2}-y_{1}\right)}^{2}}$ and $\langle \cdot \rangle$ denotes the spatial average at a given time. The self-correlation function calculates the volume fraction variation between a reference point and other spatial domains at different distances of r. Therefore, $S_{2}$ depicts the distribution of polymer-rich phases in the morphology, and the locations of the minimums in the self-correlation function correspond to the characteristic length scales of the given snapshot.

In the literature, a second approach to characterize the domain length scale is through the calculation of the average wave number for the structure factor [23,26,51,64,65,66]. In the Fourier method, the characteristic length scale is calculated as $2\pi /q_{avg}$, where the characteristic wave number is the radial average wave number of the structure factor defined as

(18) $$q_{avg}=\frac{\sum_{q}qS\left(q,t\right)}{\sum_{q}S\left(q,t\right)},$$

where the structure factor is

(19) $$S\left(q,t\right)={\left|\sum_{j_{x}=0}^{N_{x}-1}\sum_{j_{y}=0}^{N_{y}-1}[\phi \left(r,t\right){-\phi_{0}]e}^{-i\left(j_{x}\Delta xq_{x}+j_{y}\Delta yq_{y}\right)}\right|}^{2}.$$

The involved indices are consistent with those defined in Section 2.1.

## 3. Results and Discussion 

### 3.1. Phase Separation Characteristics

**Figure 2:**
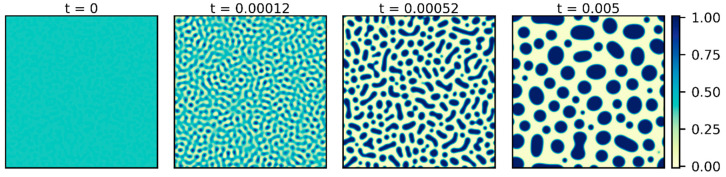
The simulation result of the evolving morphology during spinodal decomposition. The color bar shows the local polymer volume fraction. The dimensionless parameters are $\phi_{0}=0.4$, $\chi_{FH}=4$, M=1, $\kappa =7.63\times 10^{-5}$, and $m_{p}=1$. See text and Table 1 for the corresponding values in real units.

**Figure 3:**
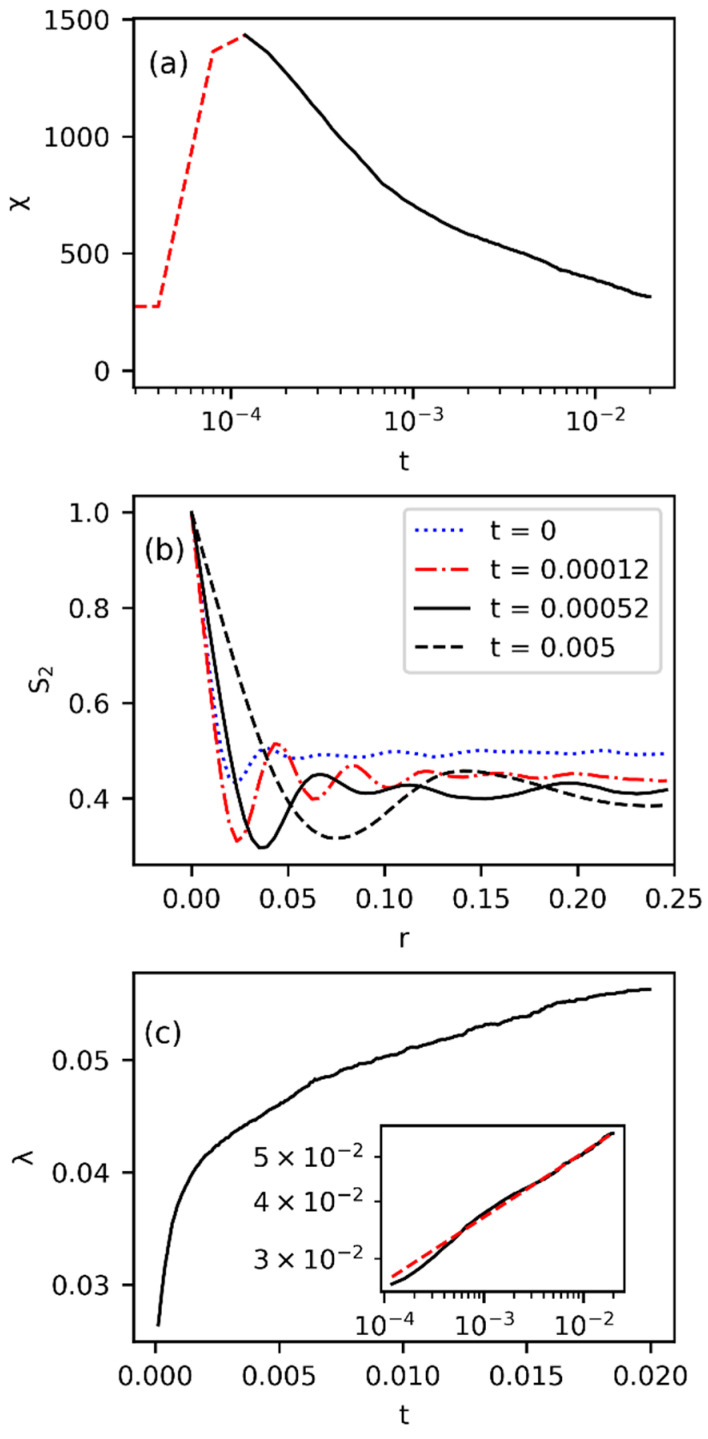
(**a**) The variation of the 2D Euler characteristic with time during phase separation. The red dashed line represents the decomposition stage, and the black solid line denotes the coarsening stage. The peak corresponds to the transition time. (**b**) The variation of the normalized self-correlation function with distance at different times. The blue curve denotes the results for the early decomposition stage, the red curve represents the correlation at the transition time, and the black curves are for the late coarsening stage. (**c**) The growth of the characteristic length during the coarsening process. The inset depicts the corresponding growth of λ in the log scale, where a reference scaling of $t^{0.141}$ is plotted in the red dashed line. The parameters are the same as in Table 1.

Figure 2 presents a typical morphology evolution during the spinodal decomposition of the polymer solution predicted by the Cahn–Hilliard equation. The initially homogeneous system demixes into regions of polymer-rich and -poor domains to form vague contours. As the concentration difference increases, the contours sharpen, and distinct phases emerge (t=0.00012). Eventually, the separated areas gradually grow in size over time (t=0.00052 and 0.005). The transition time ($t_{tr}$) of phase separation is considered the time at which a clear morphology throughout the system is formed, after which the separated phase domains grow with time. The elapsed time before $t_{tr}$ is called the decomposition stage, and the time after $t_{tr}$ is termed the coarsening stage. Consistent with the process of phase separation described, the variation of the Euler characteristic with time is shown in Figure 3a. Initially, χ remains nearly zero during decomposition since no isolated polymer-rich areas have emerged yet. When the homogeneous solution has changed to distinct phases with a morphology pattern throughout the system, an outburst of χ occurs. Eventually, the coalescence between polymer-rich domains gradually reduces the number of isolated regions, and χ decreases in the coarsening stage. 

Given the trends of χ during time evolution, we determine $t_{tr}$ as the time at which χ peaks. Therefore, the characteristic length of isolated polymer-rich phases is obtained by the location of the first minimum in $S_{2}$ at $t_{tr}$, and is named the decomposition length ($\lambda_{D}$), as shown in Figure 3b. In Figure 3b, the normalized self-correlation function starts at one and decays as the distance between two positions increases. At the transition time (red curve), $S_{2}$ shows apparent troughs and peaks with magnitudes decreasing with increased r. The oscillatory behavior characterizes the correlation of domains of the same or distinct phases compared with the reference point in space. As time increases, the coarsening effect makes the separation of neighboring peaks and troughs further apart, suggesting an increasing characteristic length of the polymer-rich phase (λ). The 2D Euler characteristic may also indicate the growing trend of the phases in terms of the decreasing connectivity during the coarsening stage. Since the total fraction of the polymer in the system is conserved, the connectivity of the polymer-rich phase decreases when the phase domains grow in size with time. The product of $\chi \lambda^{2}$ can be observed to remain constant, making $\lambda \propto \chi^{-\frac{1}{2}}$ [47]. Given the minimum characteristic length at the onset of the domain growth (also the decomposition length $\lambda_{D}$), the growing rate of the domains follows a power-law scaling, commonly given as $\lambda \propto Ct^{\alpha }$, where C is a rate constant, and α is a coarsening exponent [51]. As shown in Figure 3c, the characteristic length grows gradually in the coarsening process and displays a linear scaling on a log scale (inset), and we obtain α=0.141 after the transition time. For the characteristic length scale determined from the Fourier transform of the volume fraction distribution (Equations (18) and (19)), we obtain α=0.255, consistent with the result for the same reference system [51].

### 3.2. Effects of Parameters

After we summarize the morphological characteristics associated with the spinodal decomposition of the polymer–solvent system, we investigate the sensitivity of the system to the degree of variation of key parameters. At a prescribed temperature, five essential parameters in the Cahn–Hilliard equation include the initial fraction of the polymer ($\phi_{0}$), the mobility constant (M), the surface tension parameter (κ), the degree of polymerization ($m_{p}$), and the Flory–Huggins interaction parameter ($\chi_{FH}$). The transition time ($t_{tr}$), the decomposition length ($\lambda_{D}$), and the growth of the domain characteristic length (λ) all together elaborate the morphology complexity and the phase evolution dynamics. Therefore, we focus on analyzing the impacts of the parameters on these representative descriptors one by one. Given the reference system with dimensionless parameters of $\phi_{0}=0.4$, $\chi_{FH}=4$, M=1, $\kappa =7.63\times 10^{-5}$, and $m_{p}=1$, the degree of influence is compared for each parameter in terms of the corresponding degree of variation relative to the reference condition.

**Figure 4:**
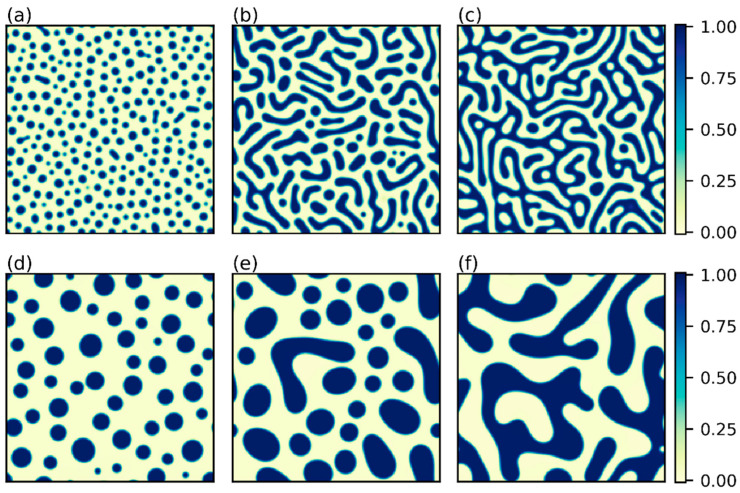
(**a**–**c**) The morphologies at t=0.0008 for $\phi_{0}=0.26$, 0.45, and 0.5, respectively. (**d**–**f**) The corresponding morphologies at t=0.012 for $\phi_{0}=0.26$, 0.45, and 0.5, respectively. Other parameters are the same as in Figure 2.

**Figure 5:**
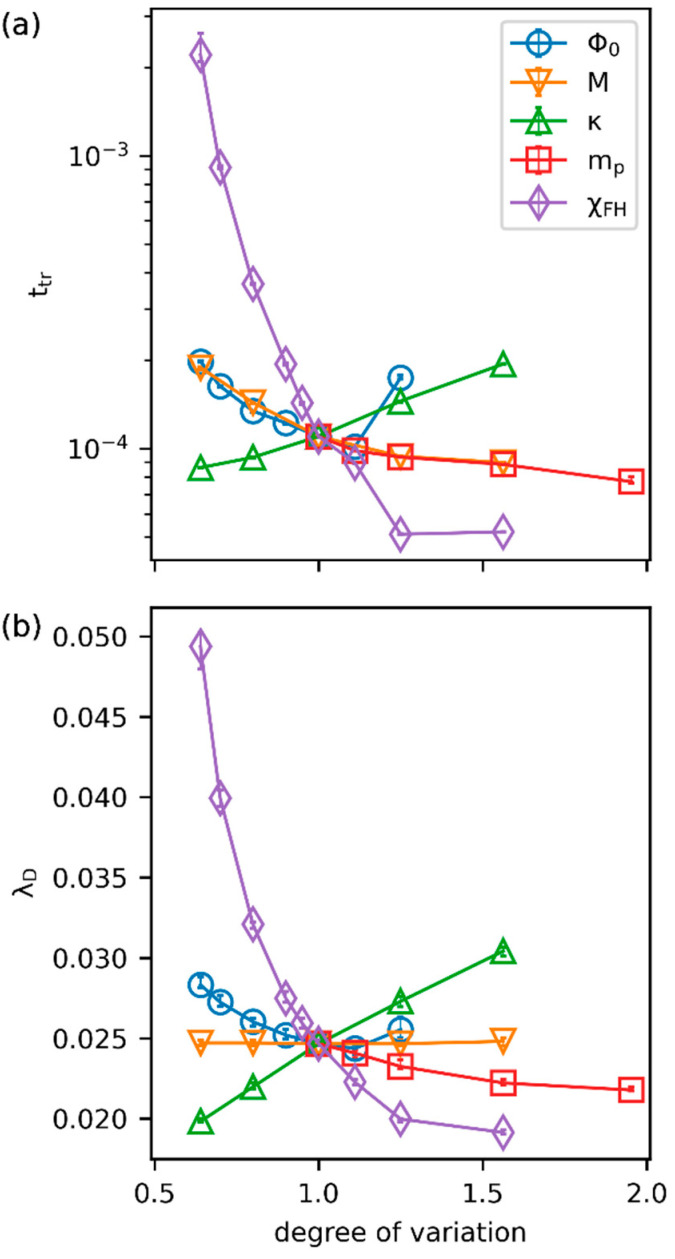
The sensitivity analysis of the five parameters to (**a**) the transition time and (**b**) the decomposition length. The degree of variation for each parameter is determined by the ratio of the parameter to its reference value. The error bars are the standard deviations of five independent simulations.

As the initial volume fraction of polymer impacts the overall fraction of polymer-rich phases in the later phase separation stage and determines the final equilibrium morphology, we first compare the system patterns for varying $\phi_{0}$ at both early and late times. As depicted in Figure 4, as $\phi_{0}$ increases, the structural feature gradually changes from spherical droplets to ellipses and eventually elongates to a tunnel-like bi-continuous pattern at $\phi_{0}=0.5$. It is anticipated that the polymer matrix will form if we further increase $\phi_{0}$ (see Figure S5d). For isolated spherical or ellipsoidal structures, a larger extent of elongation at a higher $\phi_{0}$ results in an earlier transition time. This is because at low $\phi_{0}$, species would generally be required to diffuse over a longer path to coalesce and form isolated domains. In contrast, as the polymer is concentrated enough at $\phi_{0}=0.5$, the time for a clear contour of bi-continuous morphology to form is longer, thus resulting in a later transition. A minimum of the transition time is observed roughly at $\phi_{0}=0.45$. Next, as increased mobility naturally facilitates the diffusion of species in the system, $t_{tr}$ is expected to decrease with increased M. The surface tension introduces the diffusion barrier between phases by the thickness of the interface [50]. A smaller value of κ thus alleviates the impact of the concentration gradient and reduces the distance to the interchange of species between domains. Therefore, a lower κ value also leads to a smaller $t_{tr}$. As seen in Figure S5i–l, the interfacial thickness grows with increased κ. As the degree of polymerization directly affects the size of the polymer, larger $m_{p}$ reduces the entropy gain when mixing polymer and solvent, and phase separation occurs earlier. Meanwhile, a more disfavored interaction between polymer and solvent at larger $\chi_{FH}$ triggers phase separation quickly with early-occurred sharp contours of domains. Taken together, Figure 5a demonstrates that $t_{tr}$ decreases monotonically with increased M, $m_{p}$, and $\chi_{FH}$, increases as κ is higher, and shows a minimum at $\phi_{0}$ around 0.45. 

In Figure 5b, a similar sensitivity comparison is summarized for the decomposition length. The effect of $\phi_{0}$ on $\lambda_{D}$ shows a trend that aligns with $t_{tr}$, where an earlier occurrence of phase transition results in a shorter decomposition length scale. The longer the time for polymer-rich regions to gather, the larger the domain size. Therefore, the minimum of $\lambda_{D}$ is located at $\phi_{0}=0.45$ as well. Since the equilibrium morphology is irrelevant to the diffusion rate of the species, variation of M shows negligible influence on $\lambda_{D}$. A higher surface tension parameter generates a thicker interfacial region (also see Figure S5i–l) and more considerable interfacial energy [14,50]. Therefore, the larger the κ, the longer the $\lambda_{D}$. As higher $m_{p}$ and $\chi_{FH}$ result in an early phase transition, the corresponding decomposition length is smaller. The small standard deviations presented in Figure 5 (within 0.78% of the mean for $t_{tr}$ and 0.96% of the mean for $\lambda_{D}$) suggest that the uncertainty in determining the geometrical characteristics is only minor. In Figure S6, we compare our simulated $t_{tr}$ and $\lambda_{D}$ with the theoretical predictions based on system parameters [51]. As pointed out, an arbitrary constant (we choose 6.54) is introduced to account for the difference in the definition used for $t_{tr}$. Furthermore, the two estimates for $\lambda_{D}$ based on the two approaches would be off by an $O\left(1\right)$ factor. In our calculations for the reference system at the transition time, the length scale of $2\pi /q_{avg}$ is roughly 1.71 times the location of the first minimum in $S_{2}$. The numerical $t_{tr}$ and $\lambda_{D}$ in Figure S6 have been multiplied by the corresponding two constants. The close agreement between the simulated and the theoretical results further validates our calculations of these characteristic properties.

**Figure 6:**
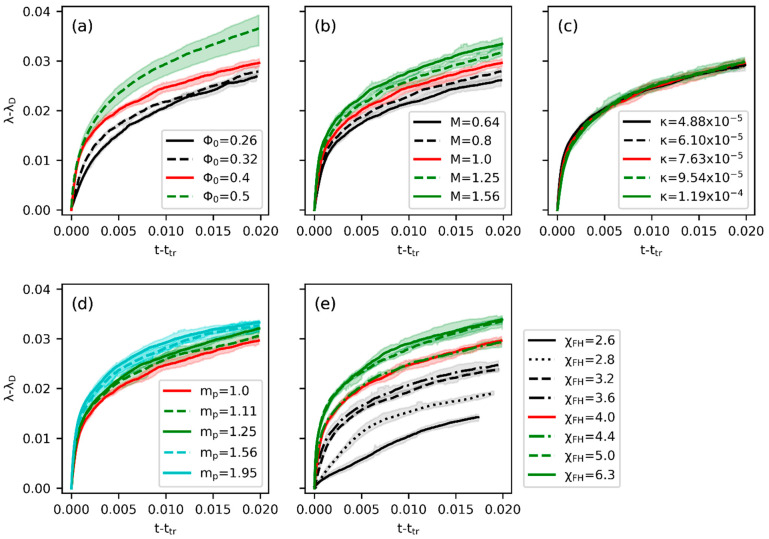
The growth of the characteristic length of the domain with time during the coarsening process for varied (**a**) initial volume fraction of polymer, (**b**) mobility constant, (**c**) surface tension parameter, (**d**) degree of polymerization, and (**e**) interaction parameter. The red solid curves correspond to the reference parameter condition. The shaded areas denote the error bounds obtained from five independent simulations.

**Figure 7:**
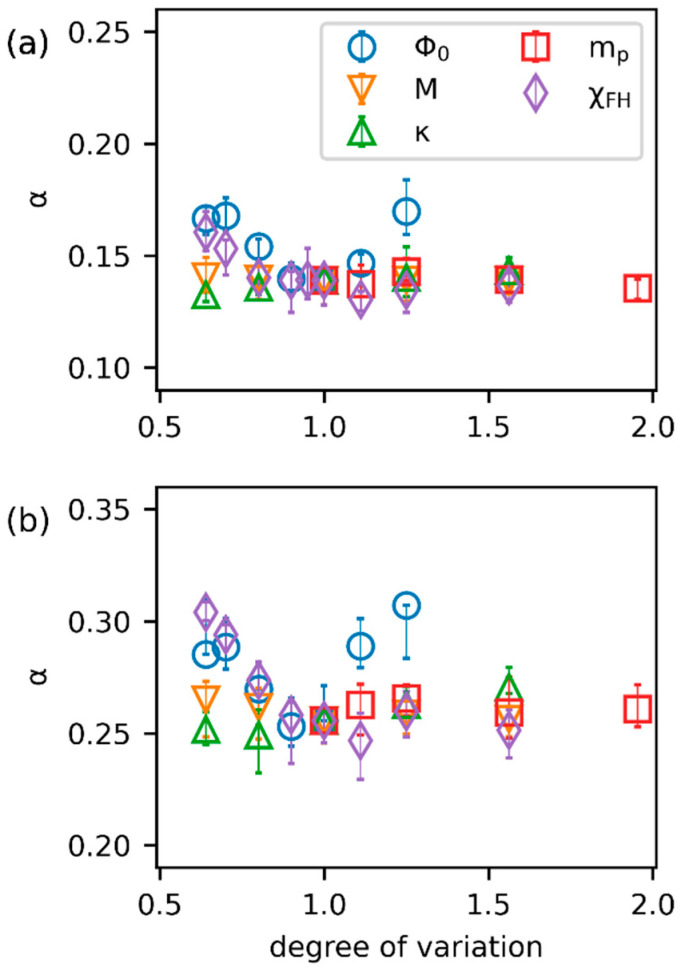
The sensitivity analysis of the five parameters to the power-law exponent for $\lambda \left(t\right)$ determined by (**a**) the current 2D Euler characteristic approach and (**b**) the average wave number of the structure factor. The degree of variation for each parameter is determined by the ratio of the parameter to its reference value. The error bars are the standard deviations of five independent simulations.

The domain growth rate is generally characterized after the transition time [51]. Therefore, in Figure 6, we compare the growth of the domain characteristic size as a function of the time increment relative to $t_{tr}$. First of all, the impact of $\phi_{0}$ on the growing characteristic length in Figure 6a may be rationalized by the morphological features presented in Figure 4. In general, the growth rate increases with the degree of connectivity between domains. Therefore, λ of the continuous structure at $\phi_{0}=0.5$ grows the fastest, while that of the droplet-like structure at $\phi_{0}=0.26$ or 0.32 grows more slowly. As the structure at $\phi_{0}=0.4$ is more elongated and tunnel-like at the early stage of the coarsening process (close to Figure 4b), the growth rate is similar to that of $\phi_{0}=0.5$. At the late stage of the coarsening process, domains coalesce into isolated ellipses, resulting in a growth law close to more dilute systems. The domain growth rate is positively influenced by increased M, $m_{p}$, and $\chi_{FH}$. A high M enhances the diffusion rate of both the solvent and polymer, thus showing a consistently increasing growth rate under different degrees of variation. Meanwhile, higher $\chi_{FH}$ and $m_{p}$ generally increase the energy state of the homogeneous system and provide a stronger driving force for coarsening. Surprisingly, the increase in λ is not sensitive to the variation of κ. This phenomenon indicates that during the coarsening stage, the free energy density (f) of distinct phases more dominantly impacts the overall domain growth rate than the interfacial energy. In Figures S7 and S8, the log-log plots of $\lambda \left(t\right)$ corresponding to Figure 6 and that obtained using the Fourier method are presented, respectively. It can be seen that the variations in the growing trends are consistent in the two figures. Figure 7 compares the associated sensitivity analyses of the power-law exponents obtained by the two approaches. Specifically in Figure 7b, the change in α with respect to $\phi_{0}$ is consistent with the results summarized by ref. [51] for symmetric mixtures ($m_{p}=m_{s}=1$) with constant mobility. At the critical composition ($\phi_{0}=0.5$; 1.25 degree of variation), a high enough $\chi_{FH}$ yields α≈1/3, as volume fraction reduces to $\phi_{0}=0.4$ (our reference system) α≈1/4, and α rises to around 1/3 again for an even smaller $\phi_{0}$. Moreover, the value of $\chi_{FH}$ at a fixed $\phi_{0}$ directly determines the quench depth ($\chi_{FH}-\chi_{c}$) for a given composition. In general, the larger the difference between $\chi_{FH}$ and $\chi_{c}$, the smaller the value of α near the critical composition ($0.4<\phi_{0}<0.6$) [51], and we obtain α≈1/4 for $3.5<\chi_{FH}<6.5$ (deeper quench) and α gradually increases to 1/3 as $\chi_{FH}$ decreases (shallower quench). The dependence of α on the variation of the other three parameters (κ, M, $m_{p}$) is relatively weak for the system with $\phi_{0}=0.4$ and $\chi_{FH}=4$. The observed trends of α determined from the 2D Euler characteristic in Figure 7a are consistent with those from the Fourier method in Figure 7b, except that the values differ for distinct approaches. In determining the domain growth rates in Figure 6, the overall standard deviations are within 10% of the mean for all cases. The broader range of errors compared with $t_{tr}$ and $\lambda_{D}$ suggests that it is more challenging to analyze the morphology during the late coarsening process precisely due to the diversity of the phase space in the same equilibrium ensemble. Nevertheless, such a broader variation of possible domain-growth paths also implies that a given evolution may be realized by a range of parameters instead of just one.

Based on the results in Figure 4, Figure 5, Figure 6 and Figure 7, we summarize that the morphological feature is mainly determined by the initial polymer volume fraction. Isolated polymer-rich phases form at low $\phi_{0}$ and spherical domains elongate gradually as $\phi_{0}$ increases. Eventually, around $\phi_{0}=0.5$, bi-continuous structures appear. If one keeps increasing $\phi_{0}$, the matrix-like morphology forms with isolated polymer-poor regions. The overall sensitivity comparisons for the early-stage transition time, decomposition length, and late-stage domain growth kinetics suggest that the Flory–Huggins interaction parameter $\chi_{FH}$ is the most influential parameter of the system. Specifically, the impact of $\chi_{FH}$ is more significant as it is closer to the critical value determined by Equation (8). Although variations of $\phi_{0}$ and $m_{p}$ affect the value of $\chi_{c}$, the corresponding degree of variation of $\chi_{c}$ is relatively small, resulting in a comparably insignificant impact on $t_{tr}$, $\lambda_{D}$, and the growth of λ. Therefore, $\chi_{FH}$ would be the morphology-determining parameter in the membrane design. Practically, selecting a “too small” $\chi_{FH}$ value may lead to a decomposition length that exceeds the desired size we aim for, and picking $\chi_{FH}$ away from the critical value makes the resulting morphology rather insensitive to any parameter variations. In the subsequent section, we prioritize the inverse design in search of $\chi_{FH}$. 

### 3.3. Inverse Prediction by PINN

**Figure 8:**
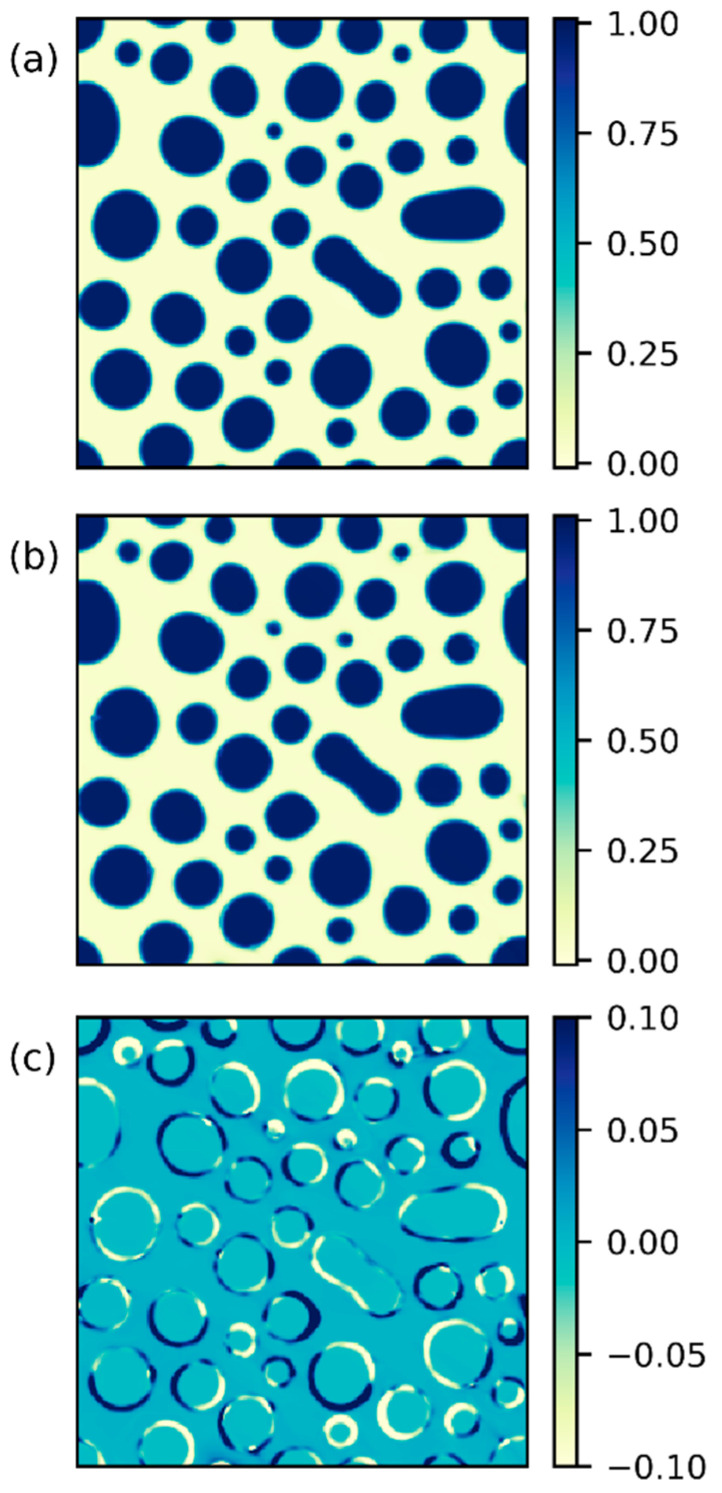
(**a**) The input morphology data for the reference system at $t_{0}=0.012$, (**b**) the corresponding PINN-learned morphology, and (**c**) the deviation (pointwise difference) between (**a**) and (**b**).

**Figure 9:**
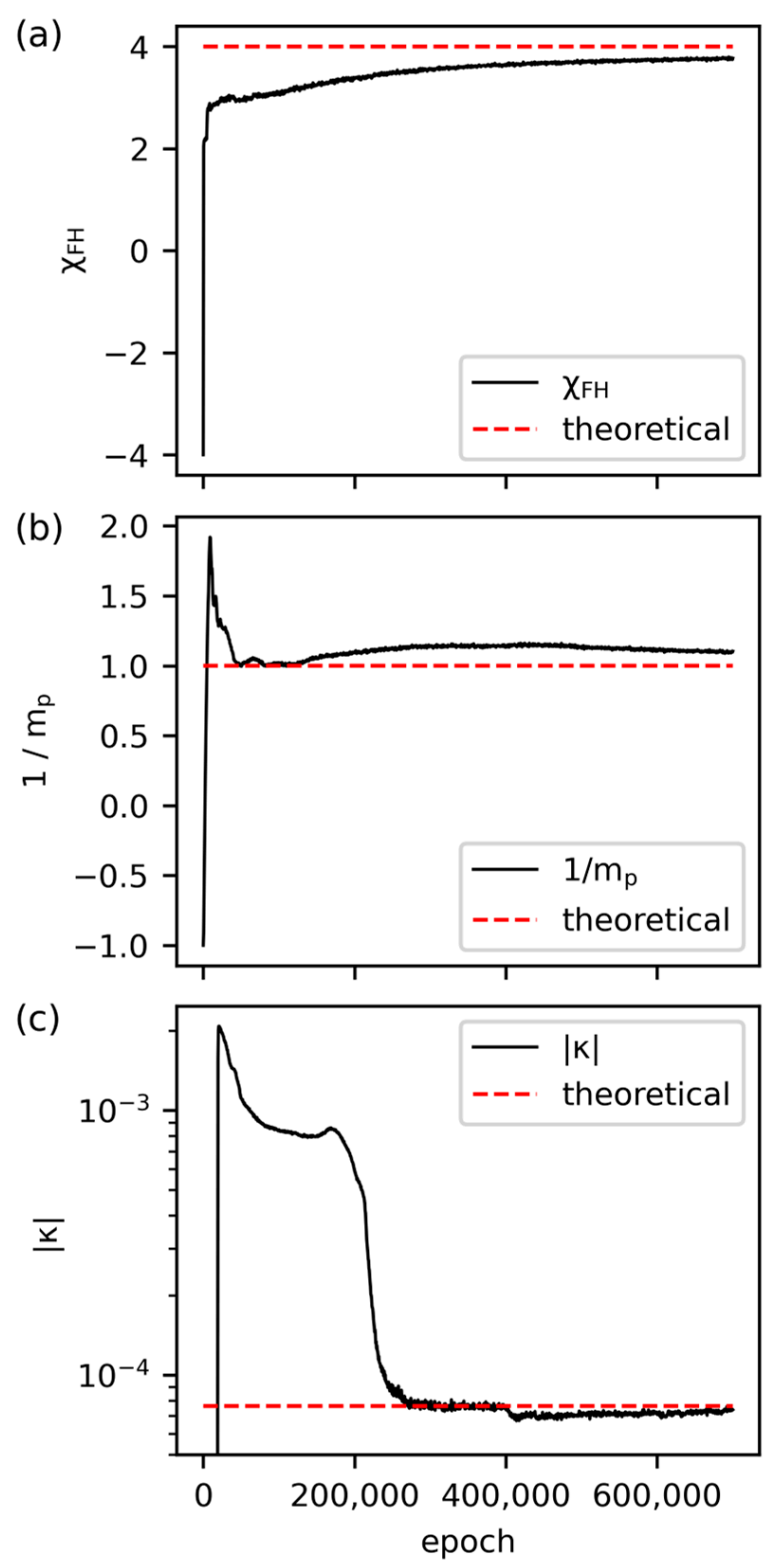
Training of the PINN in the inverse simulation. (**a**) The convergence of $\chi_{FH}$ from −4 (initial guess) to 3.83 (prediction), with an error of −4.25% relative to 4 (expected value). (**b**) The convergence of $1/m_{p}$ from −1 (initial guess) to 1.06 (prediction), with an error of −5.67% relative to 1 (expected value). (**c**) The learning curve of κ is plotted in the log scale after taking the absolute value from the initial guess of −0.1 to the predicted value of $7.39\times 10^{-5}$, −3.14% relative to the expected value of $7.63\times 10^{-5}$.

As pointed out in Section 2.2, two morphology snapshots at two distinct time steps away from the transition time are selected as input data. Fed with the data sets at $t_{0}$ and $t_{1}$, we make the chosen embedded parameter trainable and updated through iterations. In the first example, we perform inverse simulations on the reference system based on the morphologies at $t_{0}=0.012$ and $t_{1}=0.0128$ with an interval of 0.0008 (the two morphologies and their difference are shown in Figure S9a,b). As can be seen in Figure 8, the PINN is capable of capturing the complex morphology within the mean square error of 0.33% at $t_{0}=0.012$. The corresponding mean square error is 0.37% at $t_{1}=0.0128$. The small error and the pointwise deviation in Figure 8c suggest that our results are competitive with other phase-field predictions using algorithms based on convolutional neural networks [67,68]. Moreover, in Figure S9c–e, the morphological variation from $t_{0}$ and $t_{1}$ is correctly captured by PINN. Given the success in the morphology prediction, the inverse discovery of the physical parameter is also satisfactory. In Figure 9, we present the independent projections of the thermodynamic parameters that impact the energy state of the system. It reveals that the predictions of the interaction parameter, the degree of polymerization, and the surface tension parameter all yield errors within 6% of the expected values despite the large offset between the initial guess and the expected result. Based on the analysis presented in Figure 6, the growth rate shows an insignificant difference within the uncertainty range of 10%. This implies that further pursuit of higher accuracy in the predicted physical parameter may not be essential concerning a morphology design problem. Aside from the three thermodynamic parameters, the prediction of $\phi_{0}$ shall be easily accomplished through the constant overall concentration of the polymer substance. In contrast, the prediction of M is crucially coupled to the temporal scale of the input data and is not separately pursued in the inverse simulation.

We then explore the impact of the chosen time scale in morphology evolution by including more convoluted morphology patterns at earlier times as input snapshots. The results in Table 2 show that given the same interval of 0.0008, selecting morphologies at earlier times results in worse learning performance of the PINN (third column), as shown in the percentage relative error calculated by $\frac{\chi_{FH}^{ex}-\chi_{FH}^{pd}}{\chi_{FH}^{ex}}\times 100$ (fourth column), where $\chi_{FH}^{ex}=4$ represents the expected value, and $\chi_{FH}^{pd}$ is the prediction. From the comparison between the predicted morphologies at different time scales in Figure S10, the overall prediction error increases if the pattern is more convoluted with numerous small domains, and it is more challenging for the PINN to capture the concentration gradient at the phase boundaries precisely. The comparison again emphasizes that morphology complexity hinders the learnability of the PINN.

**Figure 10:**
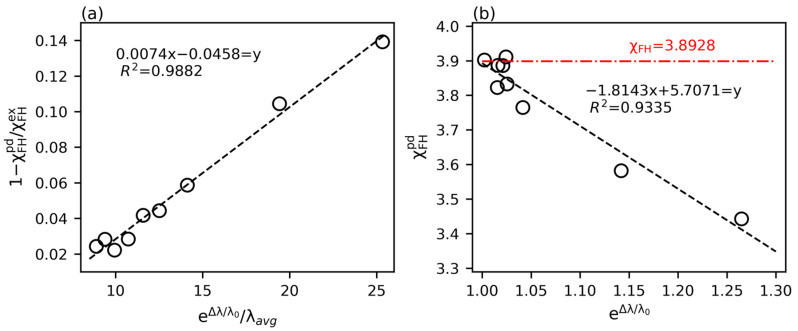
The learnability of PINN is demonstrated in terms of (**a**) the linear correlation between the relative prediction error ($1-\chi_{FH}^{pd}/\chi_{FH}^{ex}$) and the normalized morphology complexity ($e^{\Delta \lambda /\lambda_{0}}/\lambda_{avg}$) and (**b**) the linear regression of the prediction ($\chi_{FH}^{pd}$) from the morphology complexity ($e^{\Delta \lambda /\lambda_{0}}$). The red dash-dotted line indicates the extrapolated $\chi_{FH}$ at Δλ=0. The linear functions in (**a**,**b**) are the best fit with a high enough $R^{2}$.

The complexity of the morphology impacts the PINN training in two aspects. On the one hand, the complexity of the reference snapshot ($t_{0}$) introduces the inherent error in the PINN morphology prediction. On the other hand, the variation of morphology from $t_{0}$ to $t_{1}$ determines the driving potential for the PINN to relax the guessed parameter toward the optimized result. From the analysis above, we have learned that the characteristic length of the domain may represent the overall structural complexity. On a statistical basis, we choose the first minimum of the self-correlation function $S_{2}$ at the two snapshots as the structural descriptor such that $\lambda_{0}$ and $\lambda_{1}$ stand for the characteristic lengths at $t_{0}$ and $t_{1}$, respectively. A lower degree of morphological complexity apparently reduces the prediction error of the PINN. Meanwhile, the effect of structural variation should be normalized based on the average domain characteristic size of the morphology. Therefore, we quantify the morphology complexity taken by the PINN by raising the impact of morphology variation with an exponential factor normalized by the mean domain length scale:  $e^{\Delta \lambda /\lambda_{0}}/\lambda_{avg}$, where $\Delta \lambda =\lambda_{1}-\lambda_{0}$ and $\lambda_{avg}=\frac{\lambda_{0}+\lambda_{1}}{2}$, respectively. In this expression, the exponential factor characterizes the “barrier” that hinders PINN learning. The mean domain size renormalizes the impact of the barrier such that the complexity is augmented at early times with a small $\lambda_{avg}$, while the complexity is downscaled at the late stage, where $\lambda_{avg}$ is considerably large. In Figure 10a, we plot the relative error predicted by the PINN (calculated as $\left(\chi_{FH}^{ex}-\chi_{FH}^{pd}\right)/\chi_{FH}^{ex}$ or $1-\chi_{FH}^{pd}/\chi_{FH}^{ex}$) as a function of $e^{\Delta \lambda /\lambda_{0}}/\lambda_{avg}$ for the results evaluated in the time interval of 0.0008 at various coarsening stages (see Table S2). Strikingly, the data strongly correlate, and our argument is justified by the linear relation of

(20) $$1-\chi_{FH}^{pd}/\chi_{FH}^{ex}=A_{1}\left(e^{\Delta \lambda /\lambda_{0}}/\lambda_{avg}\right)+B_{1}.\;$$

In the last column of Table 2, we update the PINN prediction by substituting the corresponding value of $e^{\Delta \lambda /\lambda_{0}}/\lambda_{avg}$ into the best-fit equation. The extremely close agreement between the corrected prediction and the expected exact value proves the reliability of the linear scaling. When the exact value is given, the PINN results may be calibrated based on the linear fit to yield better predictions. In practice, $\chi_{FH}$ is an unknown parameter to be explored. By rearranging Equation (20), we arrive at another linear relation given as

(21) $$\chi_{FH}^{pd}=A_{2}e^{\Delta \lambda /\lambda_{0}}+B_{2}.$$

This new relation suggests that the PINN prediction extrapolates to the nearly exact value in the limit of ∆λ→0 such that the domain characteristic size is almost unchanged in the very late coarsening stage. In Figure 10b, we find that the linear regression extrapolates to $\chi_{FH}=3.893$ at $e^{\Delta \lambda /\lambda_{0}}=1$ labeled by the red dash-dotted line, which is merely 2.68% away from the exact value of 4. Remarkably, this extrapolated value is similar to the PINN prediction at the latest time (last row) in Table 2. Our effort in this part helps assess the accuracy of the learned parameter compared with the exact value based on the analysis of morphology complexity. Moreover, linear extrapolation correlates the predictions at different times toward a more precise result. 

## 4. Conclusions

Employing phase-field modeling using the Cahn–Hilliard equation, we have studied the morphology evolution of a binary polymer solution during phase separation in two dimensions, exemplifying the structural formation process in the membrane design. The energy of the binary mixture has been described by the Flory–Huggins theory for the homogenous state with an interaction parameter, and the effect of the concentration gradient is taken into account through a surface tension parameter. A two-way strategy for examining the morphology properties has been pursued. In the forward approach, the temporal evolution and the spatial variation of the polymer distribution have been predicted by solving the governing equation numerically using the pseudo-spectral technique. The phase-separating morphologies are characterized through the geometrical 2D Euler characteristic for the domain connectivity and the statistical self-correlation function of the polymer distributions. Using these descriptors, we have analyzed the transition time, decomposition length, and domain growth rate. The impact of each system parameter on the morphology properties has been explained through our sensitivity comparison. Among the five essential parameters, namely the initial polymer concentration, the mobility, the surface tension parameter, the degree of polymerization, and the Flory–Huggins interaction parameter, the last one appears to be the most crucial to the evolution dynamics and is termed the morphology-determining parameter. 

Complementing the forward approach, we have devised a simple-structured physics-informed neural network (PINN) to search for the embedded interaction parameter in the polymer/solvent system. On the one hand, the PINN is capable of solving the governing Cahn–Hilliard equation with the supply of necessary physical constraints. On the other hand, we have demonstrated that the network parameters can be optimized in search of the embedded parameter space associated with the input data incorporating morphology variation. Strikingly, the error of the predicted parameter relative to the expected actual value is bounded by the uncertainty in determining the domain growth rate. Taking the Flory–Huggins interaction parameter as the primary unknown parameter, we have obtained a satisfactory linear correlation between the learnability of the PINN and the degree of complexity of the fed morphology data. It suggests that the relative error of PINN prediction is proportional to an exponential factor of the degree of domain size variation; the more significant the structural changes during training, the larger the prediction error. Through transforming the correlation, the ultimate predicted parameter can be accessed by extrapolating the regression line to a zero structural variation. Such an inverse approach is envisioned to be of great use when the embedded unknown parameter for a specific system is to be discovered. Furthermore, by having the targeted membrane morphologies as input data, the interactions between the membrane constituents and their compositions may be tailored by the versatile inverse simulation.

## Figures and Tables

**Table 1:** The reference system parameters in real units.

System Parameters		Value
initial polymer volume fraction	$\phi_{0}$	0.4
system temperature	T*	300 K
unit volume of a lattice	*ν*_0_ *	10^−3^ m^3^ mol^−1^
gas constant	*R*	8.314 J K^−1^ mol^−1^
degree of polymerization (polymer)	*m_p_*	1
degree of polymerization (solvent)	*m_s_*	fixed as 1
Flory–Huggins parameter	*χ_FH_*	4
surface tension parameter	κ *	2 × 10^−10^ J m^−1^
mobility constant	M*	4 × 10^−17^ m^5^ s^−1^ J^−1^
system domain	L*	1024 nm
time	t*	0.01 s

**Table 2:** Representative PINN-predicted values of $\chi_{FH}$ at different time stages with the same interval.

*t*_0_	*t*_1_	$\mathrm{Predicted}\;\chi_{FH}$	Relative Error (%)	$\mathrm{Corrected}\;\chi_{FH}$
0.0008	0.0016	3.44	13.92	4.01
0.0016	0.0024	3.58	10.45	3.97
0.004	0.0048	3.76	5.87	4.00
0.0152	0.016	3.91	2.21	4.02

## Data Availability

The data and codes supporting the findings of this study are available from the corresponding author upon reasonable request.

## Supplementary Materials

The following supporting information can be downloaded at: https://www.mdpi.com/article/10.3390/polym15244711/s1, Table S1: PINN predictions with different weights of the loss terms; Figure S1. Training of the PINN in the inverse simulation for five weight choices in Table S1. Case 1 (solid cyan) corresponds to the result in Figure 9a; Figure S2: Training of the PINN in the inverse simulation for different learning rates (lr). The solid cyan line corresponds to the result in Figure 9a; Figure S3: Training of the PINN in the inverse simulation for different numbers of initially sampled collocation points. The solid cyan line corresponds to the result in Figure 9a; Figure S4: Training of the PINN in the inverse simulation for different numbers of newly sampled collocation points for each epoch. $N_{PDE}=0$ corresponds to the case without additional sampling for each epoch. The solid cyan line corresponds to the result in Figure 9a; Figure S5: Simulated morphologies at t=0.02 for the reference system with one parameter varied: (a–d) $\phi_{0}=0.26$, 0.32, 0.5, and 0.6; (e–h) M=0.64, 0.8, 1.25, and 1.5; (i–l) κ=4.88, 6.1, 9.53, and $11.8\left(\times 10^{-5}\right)$; (m–p) $m_{p}=1$, 1.25, 1.56, and 1.95; (q–t) $\chi_{FH}=2.5$, 3.2, 5, and 6.3. Other parameters are the same as in Figure 2; Figure S6: (a) Simulated transition time versus theoretical transition time and (b) simulated decomposition length versus theoretical decomposition length for a given varied parameter; Figure S7: The log-log plots of $\lambda \left(t\right)$ for given varied parameters corresponding to Figure 6 without time and length shifts. The lines are multiplied with some arbitrary constants for a better visualization; Figure S8: The log-log plots of $\lambda \left(t\right)$ for given varied parameters obtained from the Fourier method described in the text. The lines are multiplied with some arbitrary constants for a better visualization; Figure S9: The input morphologies for the reference system at (a) $t_{0}=0.012$ and (b) $t_{1}=0.0128$. (c) The variation between (a,b). (d) The variation between the PINN-learned morphologies at $t_{0}=0.012$ and $t_{1}=0.0128$. (e) The difference between (c,d); Figure S10: (a) The input morphology data at t=0.0024, (b) the corresponding PINN-learned morphology, and (c) the deviation between (a,b). (d) The input morphology data at t=0.02, (e) the corresponding PINN-learned morphology, and (f) the deviation between (d,e); Table S2: Data and PINN predictions at different time stages with the same interval of 0.0008.
