# Supplementary material for: Connecting Structural Characteristics and Material Properties in Phase-Separating Polymer Solutions: Phase-Field Modeling and Physics-Informed Neural Networks
Source: Polymers (Basel). 2023 Dec 14;15(24):4711. doi: 10.3390/polym15244711 (PMC10748238; doi:10.3390/polym15244711)
Supplement: Supplementary file 1 [file polymers-15-04711-s001.zip › polymers-2671050-supplementary.pdf]

Supplementary Information for:

Connecting Structural Characteristics and Material Properties in Membrane-Forming Polymers: Phase-Field Modeling and Physics-Informed Neural Networks

Le-Chi Lin, Sheng-Jer Chen, and Hsiu-Yu Yu[*]

Department of Chemical Engineering, National Taiwan University,

No. 1, Sec. 4, Roosevelt Rd., Taipei 10617, Taiwan

E-mail: hsiuyuyu@ntu.edu.tw

Table S1. PINN predictions with different weights of the loss terms.

	J_{PDE}	J_{t_0}/J_{t_1}	J_{BC}	J_{μ}	χ_{FH}
Case 1	1	100	1	10	3.83
Case 2	100	1	1	10	3.7
Case 3	1	1	1	1	3.81
Case 4	1	1	1	100	3.1
Case 5	1	1	100	1	3.79

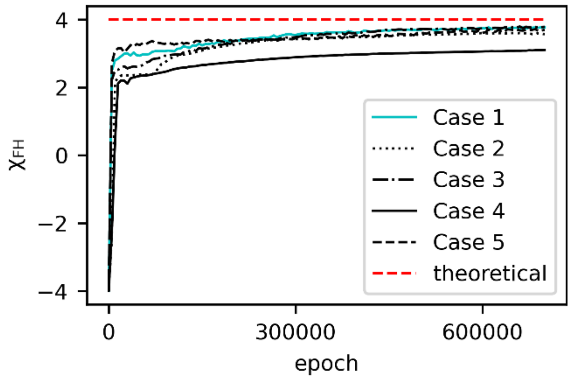

Figure S1. Training of the PINN in the inverse simulation for five weight choices in Table S1. Case 1 (solid cyan) corresponds to the result in Figure 9(a).

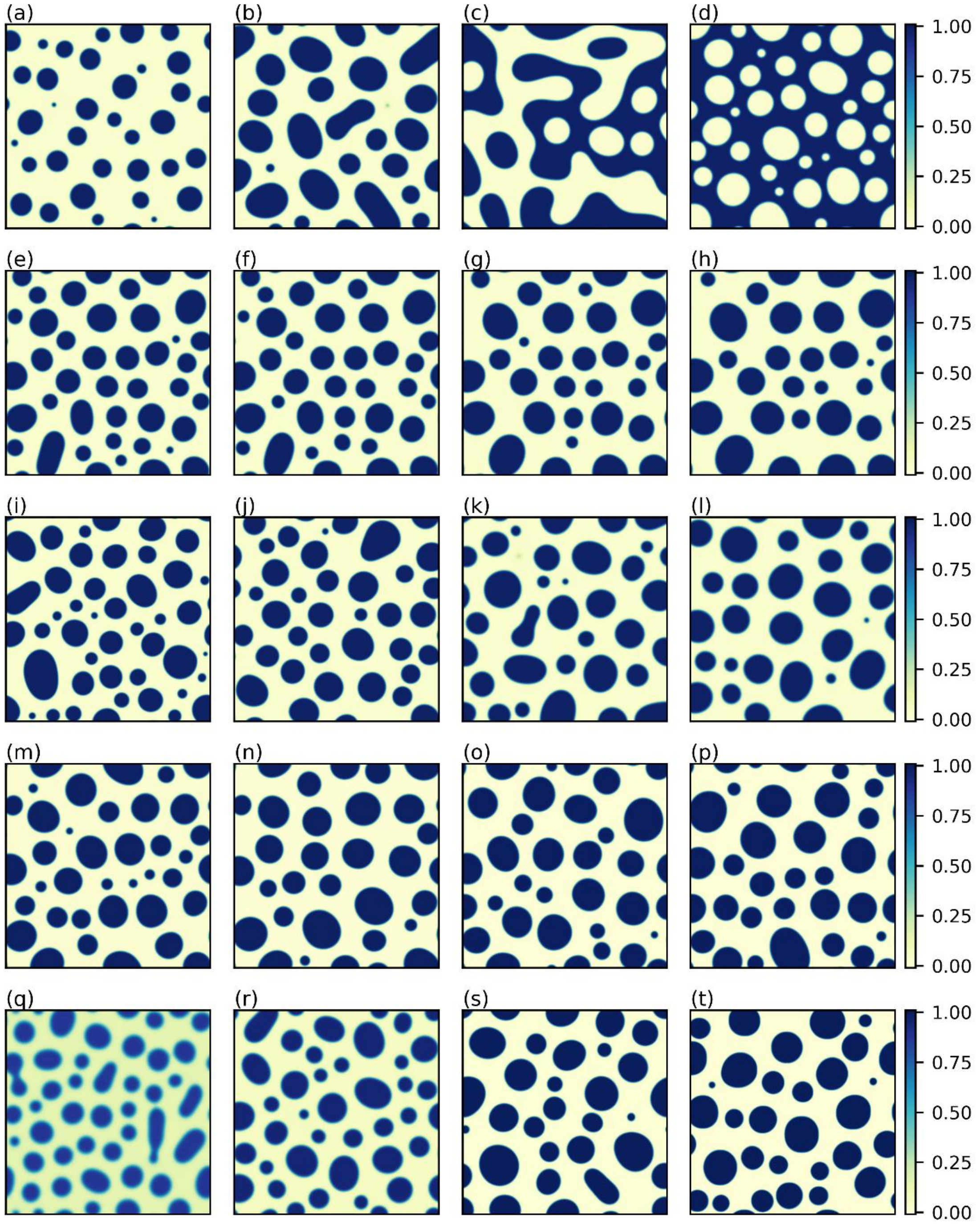

Figure S2. Simulated morphologies at $t = 0.02$ for the reference system with one parameter varied: (a)-(d) $\phi_0 = 0.26,\ 0.32,\ 0.5$, and 0.6; (e)-(h) $M = 0.64,\ 0.8,\ 1.25$, and 1.5; (i)-(l) $\kappa = 4.88,\ 6.1,\ 9.53$, and $11.8\ (\times 10^{-5})$; (m)-(p) $m_p = 1,\ 1.25,\ 1.56$, and 1.95; (q)-(t) $\chi_{FH} = 2.5,\ 3.2,\ 5$, and 6.3. Other parameters are the same as in Figure 2.

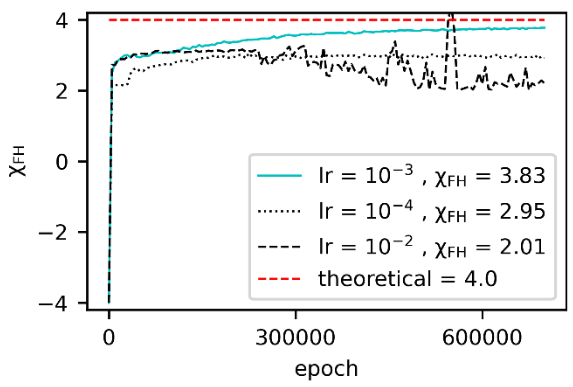

Figure S3. Training of the PINN in the inverse simulation for different learning rates (lr). The solid cyan line corresponds to the result in Figure 9(a).

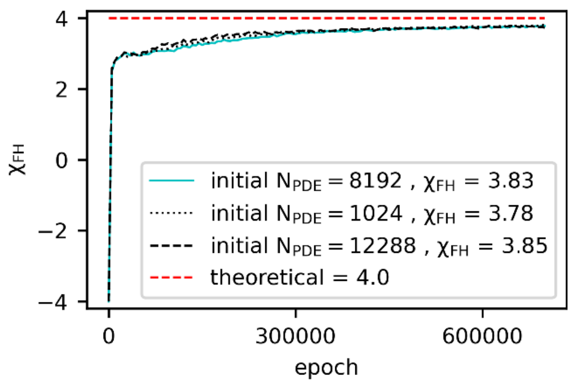

Figure S4. Training of the PINN in the inverse simulation for different numbers of initially sampled collocation points. The solid cyan line corresponds to the result in Figure 9(a).

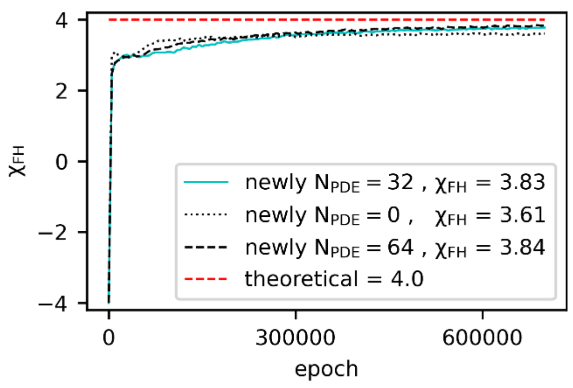

Figure S5. Training of the PINN in the inverse simulation for different numbers of newly sampled collocation points for each epoch. $N_{PDE} = 0$ corresponds to the case without additional sampling for each epoch. The solid cyan line corresponds to the result in Figure 9(a).

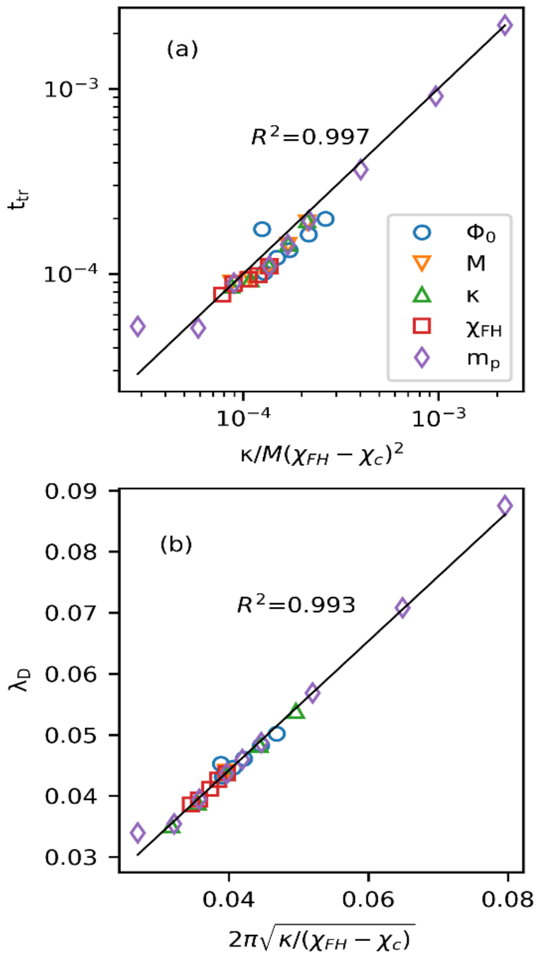

Figure S6. (a) Simulated transition time versus theoretical transition time and (b) simulated decomposition length versus theoretical decomposition length for a given varied parameter.

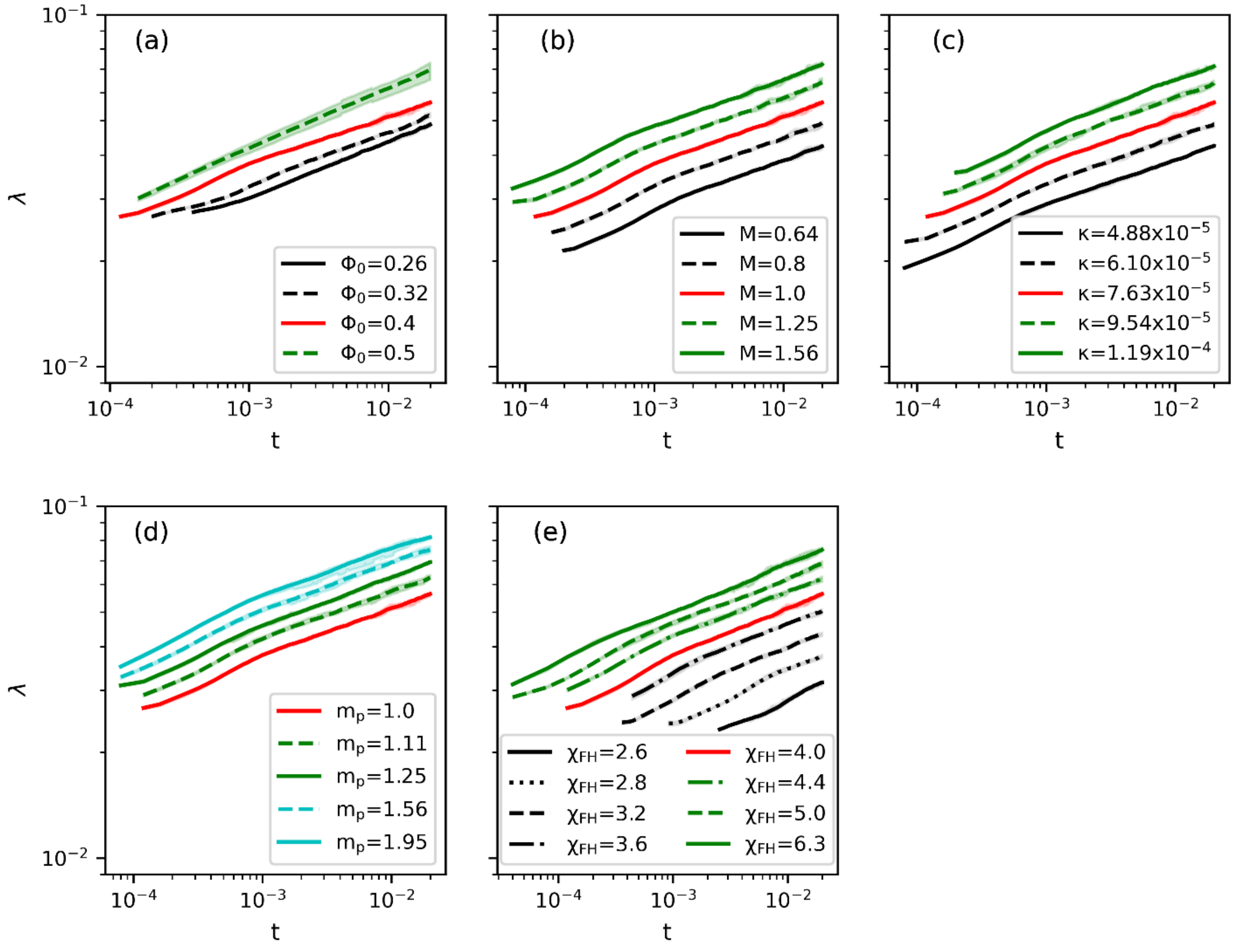

Figure S7. The log-log plots of $\lambda(t)$ for given varied parameters corresponding to Figure 6 without time and length shifts. The lines are multiplied with some arbitrary constants for a better visualization.

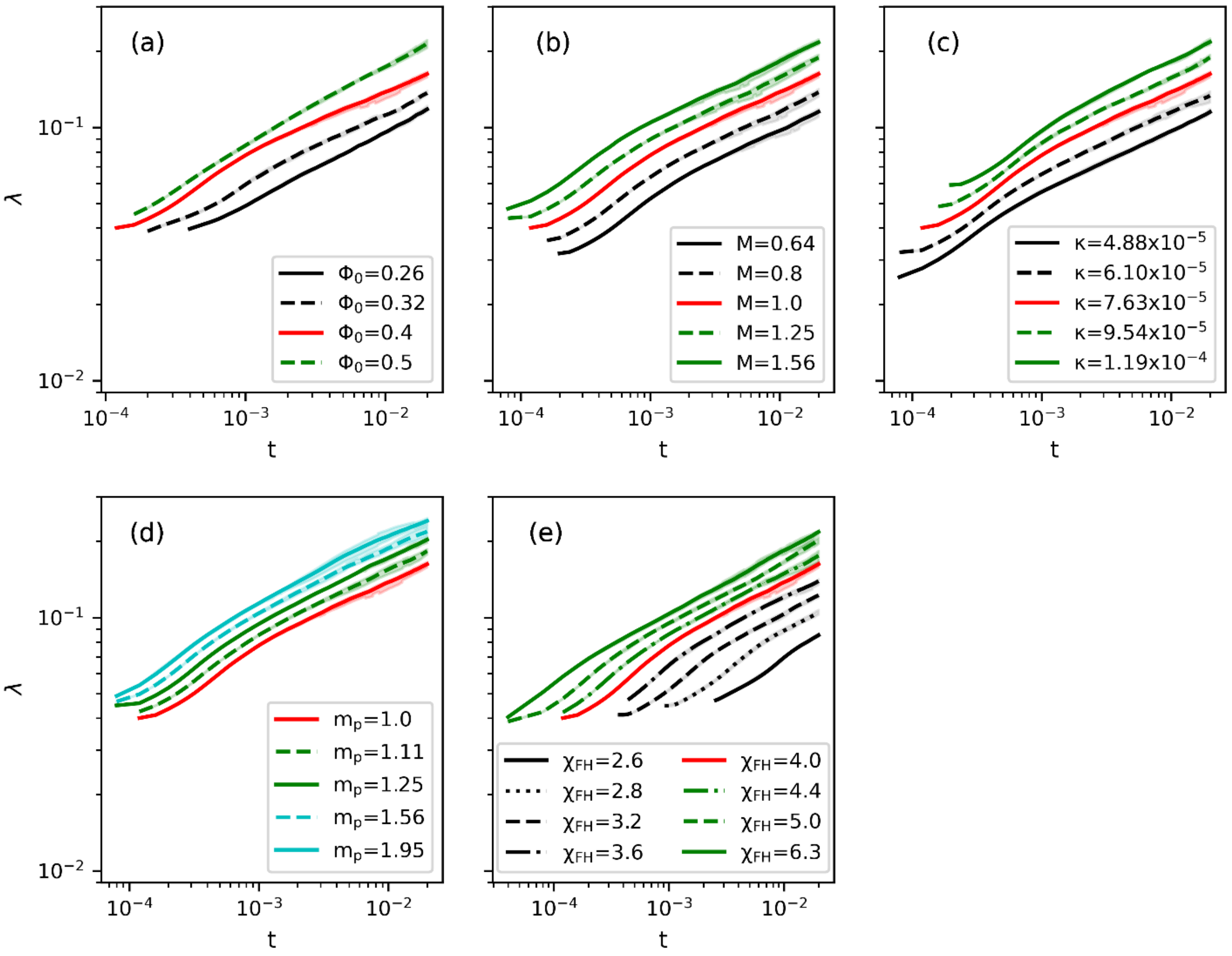

Figure S8. The log-log plots of $\lambda(t)$ for given varied parameters obtained from the Fourier method described in the text. The lines are multiplied with some arbitrary constants for a better visualization.

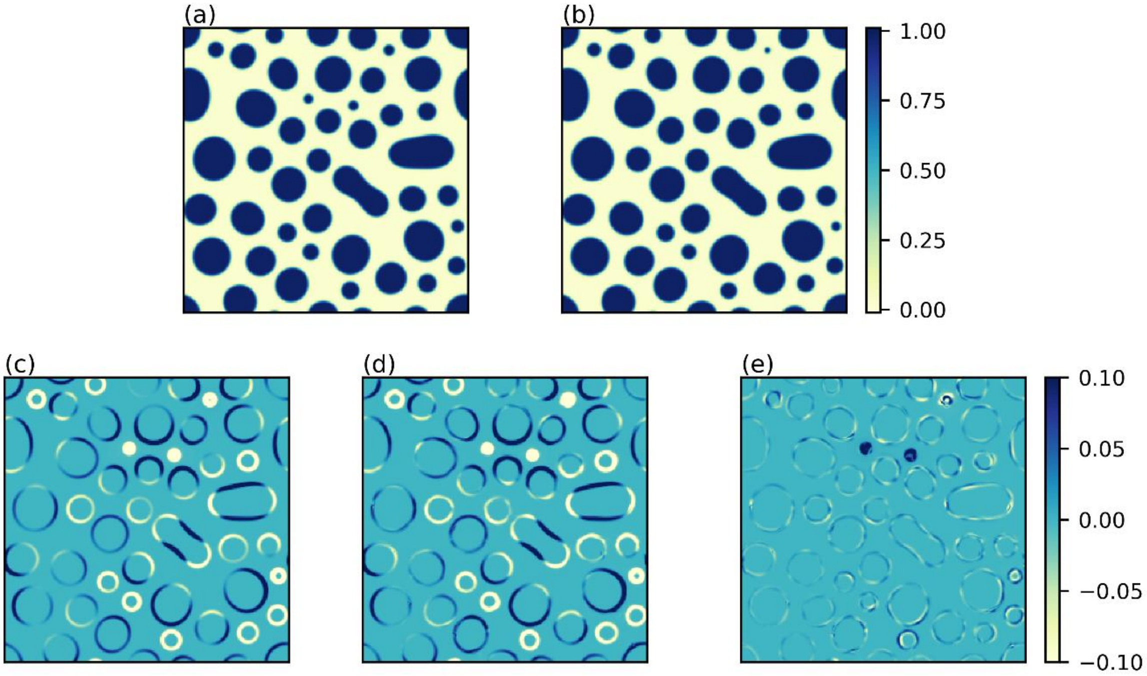

Figure S9. The input morphologies for the reference system at (a) $t_0 = 0.012$ and (b) $t_1 = 0.0128$. (c) The variation between (a) and (b). (d) The variation between the PINN-learned morphologies at $t_0 = 0.012$ and $t_1 = 0.0128$. (e) The difference between (c) and (d).

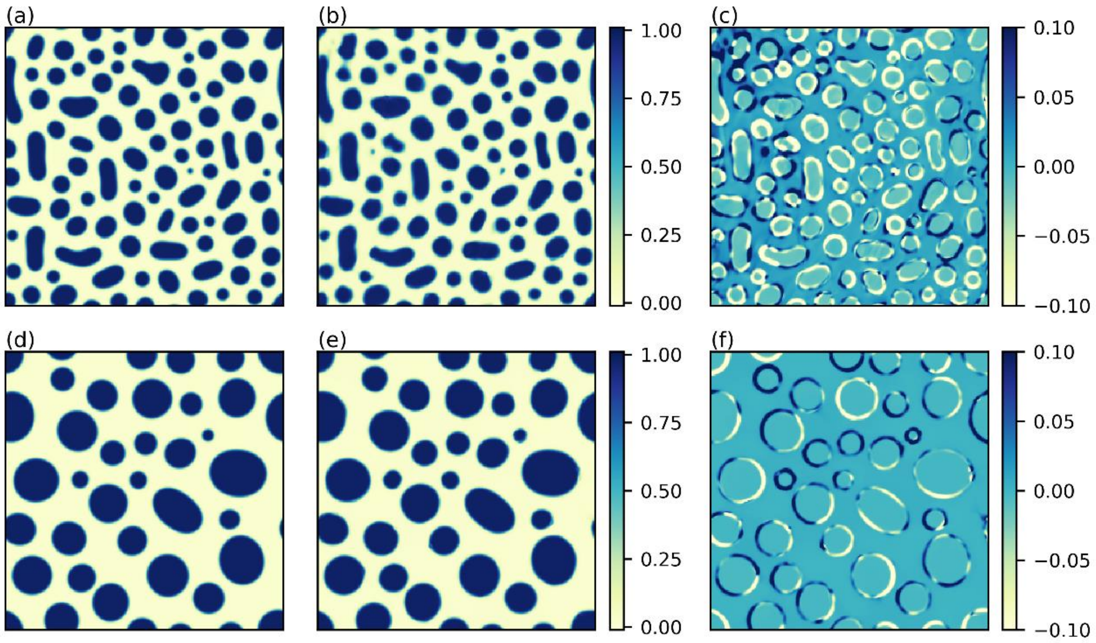

Figure S10. (a) The input morphology data at $t = 0.0024$, (b) the corresponding PINN-learned morphology, and (c) the deviation between (a) and (b). (d) The input morphology data at $t = 0.02$, (e) the corresponding PINN-learned morphology, and (f) the deviation between (d) and (e).

Table S2. Data and PINN predictions at different time stages with the same interval of 0.0008.

t_0	t_1	S_2 at t_0	S_2 at t_1	predicted χ_{FH}	relative error (%)	corrected χ_{FH}[a]
0.0008	0.0016	0.0447	0.0552	3.44	13.92	4.01
0.0016	0.0024	0.0551	0.0625	3.58	10.45	3.97
0.004	0.0048	0.0722	0.0752	3.76	5.87	4.00
0.0064	0.0072	0.0805	0.0818	3.82	4.43	4.01
0.0096	0.0104	0.0874	0.0896	3.83	4.17	3.99
0.0128	0.01136	0.0942	0.0962	3.88	2.85	4.02
0.0152	0.016	0.1018	0.1042	3.91	2.22	4.02
0.0176	0.0184	0.1074	0.1091	3.88	2.83	3.98
0.02	0.0208	0.1125	0.1127	3.90	2.44	3.98

[a]. Corrected values are obtained from the correlation line in Figure 10(a).
